# Hydrogen‐deuterium exchange reveals catalytically linked protein flexibility in myoglobin‐mediated intramolecular C(sp^3^)‐H activation

**DOI:** 10.1002/pro.70410

**Published:** 2025-12-22

**Authors:** Hanzi Gao, Edgar Africano Camargo, Jude N. Ubi, Xiuyuan Duan, Xiaolin Tian, Haiteng Deng, Guojun Zheng, Shuaihua Gao

**Affiliations:** ^1^ State Key Laboratory of Chemical Resources Engineering Beijing University of Chemical Technology Beijing China; ^2^ Department of Chemical and Biomolecular Engineering Tulane University New Orleans Louisiana USA; ^3^ MOE Key Laboratory of Bioinformatics, School of Life Sciences Tsinghua University Beijing China

**Keywords:** conformational landscape, hydrogen‐deuterium exchange (HDX), protein dynamics myoglobin, protein flexibility

## Abstract

A comprehensive understanding of the biophysical parameters that dictate high catalytic efficiency in enzymes is essential for advancing both fundamental enzymology and its applications. Experimental evidence suggests that protein dynamics play a pivotal role in transiently shaping active site configurations, facilitating the efficient traversal of reaction barriers. In a previous study, protein engineering led to the development of a triple mutant of myoglobin, which enabled the successful synthesis of an array of chiral flavanone compounds through myoglobin‐mediated carbene transfer. To gain deeper insights into the molecular mechanisms underlying the evolution of structural dynamics that contribute to the accelerated catalytic properties, we first performed hydrogen‐deuterium exchange mass spectrometry (HDX‐MS) analyses on both the wild‐type myoglobin and the engineered triple mutant in the presence and absence of a substrate analog to elucidate conformational changes and impacts of mutations on protein flexibility and functional dynamics. HDX‐MS analysis identified distinct regions of the protein, both proximal and distal to the mutation sites, which exhibited differential HDX behaviors in response to either ligand binding or mutation, thereby providing insights into the structural and dynamic evolution of the mutant. We postulate that the mutation reconfigures the conformational ensemble of the protein, thereby promoting favorable conformational sampling and enhancing the efficiency of the catalyzed reaction. Computational studies further support this conclusion, providing additional insights into the structural and dynamic factors influencing enzymatic efficiency. This study highlights the critical role of protein structural dynamics in evolved enzymes, underscoring the potential of probing and harnessing these dynamics for advancements in protein engineering and redesign.

## 1 INTRODUCTION

Unlike static structures, proteins are inherently dynamic, constantly undergoing conformational fluctuations that are essential to their function (Henzler‐Wildman and Kern 2007; Klinman 2015; Lewandowski et al. 2015; Tousignant and Pelletier 2004). The dynamic nature of proteins enables them to perform catalysis with precision for diverse reactions with enormous rate acceleration (Lipscomb 1982; Radzicka and Wolfenden 1995). Protein motions occur across a wide range of timescales, spanning from rapid picosecond‐to‐nanosecond fluctuations to much slower millisecond‐to‐second conformational rearrangements (Henzler‐Wildman et al. 2007). Larger conformational changes can be readily observed using techniques like X‐ray crystallography (Keedy et al. 2015b), cryo‐electron microscopy (Danev et al. 2019), or nuclear magnetic resonance (NMR) spectroscopy (Mittermaier and Kay 2006). These subtle conformational changes involve small, localized structural movements. Individually modest, they collectively modulate the protein's overall conformational landscape (Damry et al. 2019; Nussinov et al. 2023; Ono et al. 2023). These dynamic fluctuations play a crucial role in regulating protein function by influencing molecular recognition, ligand binding, allosteric communication, and catalytic efficiency (Jia et al. 2020; Minshull et al. 2024; Woods et al. 2024; Zhu et al. 2024).

Understanding these nuanced structural shifts offers important insight into protein stability, flexibility, and activity. Such insight underpins advances in enzymology, structural biology, and protein engineering and design. However, unlike large‐scale conformational transitions, these subtle structural changes are often challenging to detect using conventional techniques such as X‐ray crystallography (DePristo et al. 2004), because their transient nature and low‐amplitude motions make them unlikely to appear in static crystal structures. These techniques generally yield an ensemble‐averaged conformation rather than revealing dynamic movements. As a result, alternative approaches, such as time‐resolved X‐ray crystallography (Levantino et al. 2015; Šrajer and Schmidt 2017; Van Den Bedem et al. 2013; Yabukarski et al. 2020), nuclear magnetic resonance (NMR) spectroscopy (Otten et al. 2010; Stiller et al. 2022; Wolf‐Watz et al. 2004), hydrogen‐deuterium exchange mass spectrometry (HDX‐MS) (Englander 2006; Gao et al. 2020; Gao et al. 2022; Gao and Klinman 2022; Masson et al. 2019), and molecular dynamics (MD) simulations (Allison 2017; Karplus and Kuriyan 2005; Martin and Frezza 2022; Wang et al. 2024), are often required to probe these fine‐scale conformational dynamics with higher temporal and spatial resolution (Keedy et al. 2015a).

Among these analytical techniques, HDX‐MS has emerged as a powerful tool for investigating protein dynamics (Marcsisin and Engen 2010; Offenbacher et al. 2017; Ozohanics and Ambrus 2020; Wales and Engen 2006). This method provides high spatial resolution detection of localized conformational changes and dynamic fluctuations across different regions of a protein (James et al. 2021) by accurately measuring backbone amide hydrogen exchange rates. This makes HDX‐MS an invaluable technique for elucidating protein conformational landscapes and their functional implications in enzymatic activity, allostery, and ligand binding. In this approach, the deuteron incorporation into backbone amides of a protein is monitored as a function of time and temperature, undergoing quenching of protein samples and their subsequent digestion into small peptides followed by high performance liquid chromatography‐mass spectrometry (HPLC‐MS) detection for mass detection (Konermann et al. 2011; Zhang et al. 2020a). This results in a library of peptides corresponding to the protein sequence that can be used for detailed study on the dynamic nature of each region of the protein.

In recent years, substantial progress has been achieved in hemoglobin‐ and myoglobin‐mediated carbene transfer reactions, marking a paradigm shift in biocatalysis from exploiting native functions to engineering artificial catalytic capabilities (Brandenberg et al. 2017; Brandenberg et al. 2019; Coelho et al. 2013; Davies and Manning 2008; Ortiz de Montellano 2010; Poulos 2014; Singh et al. 2014). Native globins such as myoglobin function primarily as oxygen storage proteins and lack inherent chemical reactivity. To confer catalytic function, protein engineering efforts have consistently targeted the distal histidine residue, frequently substituting it with residues such as valine, thereby generating an entry point for carbene transfer activity (Paoli et al. 2002; Reedy and Gibney 2004). The reaction mechanisms and key catalytic intermediates of these engineered heme proteins have since been elucidated in detail (Carminati and Fasan 2019; Prier et al. 2017; Wei et al. 2017; Zhang et al. 2019; Zhang et al. 2020b), enabling the development of innovative platforms for sustainable chemical synthesis. In our work, we have engineered myoglobin variants to catalyze the intramolecular C(sp^3^)‐H functionalization of diazo compounds, affording chiral flavanones, versatile intermediates in the synthesis of pharmacologically active compounds and therapeutic agents (Falcone Ferreyra et al. 2012; Meng and Wang 2016; Nibbs and Scheidt 2012). The initial active variant, H64V, exhibited basal carbene transfer activity and is hereafter referred to as WT′, distinguishing it from wild‐type myoglobin, which serves as a storage protein devoid of chemical reactivity. Iterative rounds of protein engineering yielded a triple mutant (V64Q/T67I/A68G) with markedly enhanced activity (Gao et al. 2025) (Figure S1, Supporting Information and Table S1). The resulting chiral flavanones possess diverse biological activities, including anti‐inflammatory, antioxidant, and anticancer properties, underscoring their value in drug discovery and development (Mutha et al. 2021; Panche et al. 2016; Reetz 2019; Safe et al. 2021; Tungmunnithum et al. 2018).

**FIGURE 1:**
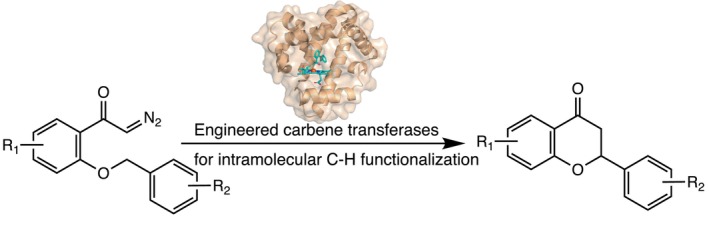
Myoglobin variants catalyzed C(sp^3^)‐H activation for stereoselective intramolecular cyclization of diazo substrates via carbene transfer activity.

In this study, we employ WT′ myoglobin and its engineered triple mutant as model systems to dissect the interplay between protein conformational dynamics and catalytic function (Gao et al. 2025) (Figure 1). By integrating hydrogen‐deuterium exchange mass spectrometry (HDX‐MS) with computational analyses, we aim to elucidate how specific mutational perturbations and ligand interactions reshape the conformational landscape of the protein, thereby facilitating enhanced catalytic activity. These mechanistic insights will provide a molecular‐level framework for understanding how engineered mutations redirect structural dynamics to enable non‐natural enzymatic transformations.

## 2 RESULTS

### 2.1 Construction of non‐overlapping peptide set for myoglobin to map spatial resolution HDX‐MS

Hydrogen‐deuterium exchange mass spectrometry (HDX‐MS) is increasingly utilized to address a broad range of biological questions, providing valuable insights into protein–protein interactions (Zhu et al. 2024), protein–ligand interactions (Minshull et al. 2024), conformational changes (Jia et al. 2020), and allosteric regulation (Woods et al. 2024). The HDX reaction involves an exchange of covalently bonded peptidyl backbone amide protons by solvent deuterons, at a rate that is influenced by inductive and steric blocking effects, with the latter being primarily a consequence of a given protein's secondary and tertiary structure (Gao et al. 2020). For a general HDX experiment the course of deuteron incorporation into backbone amides is monitored as a function of time, undergoing time‐dependent quenching of protein samples and their subsequent digestion into small peptides. A representative subset of peptides from the HDX experiment is used to visualize the behavior of the protein. Though X‐ray structures show proteins to be densely packed, native proteins in solution are known to exist in a dynamic equilibrium between locally open (*k*_open_) and closed states (*k*_close_), via a natural “breathing” process (Gao et al. 2020; Gao et al. 2022). During HDX, transient and reversible openings of backbone hydrogen bonds expose the amide N‐H groups to D_2_O, allowing intrinsic exchange (*k*_int_) to occur. In general, under native conditions, the observed rate of HDX (*k*_HDX_) is studied within an EX‐2 regime (*k*_int_ ≪ *k*_close_, such that there are numerous local unfolding and refolding events before the amide will exchange with solvent). Thus, the observed *k*_HDX_ is the product of the equilibrium constant between the local open and closed states (*K*_op_ = *k*_open_/*k*_close_) multiplied by the intrinsic HDX process (*k*_int_): *k*_HDX_ = *K*_op_ *k*_int_. When HDX experiments on two proteins with minimal amino acid sequence difference were performed, comparisons of HDX changes primarily result from the equilibrium constant difference reflecting protein structural dynamics changes within these two targeted proteins.

**FIGURE 2:**
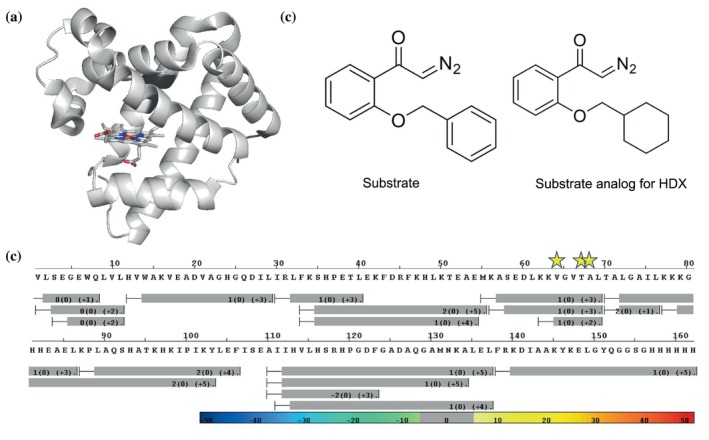
Protein sequence coverage map of myoglobin and ligands used for HDX‐MS. (a) Overlapping peptides are mapped onto the myoglobin structure. Residues detected by HDX‐MS are highlighted in light gray, whereas undetected residues are shown in dark gray. (b) Sequence coverage map for the overlapping peptides. Mutation sites are indicated with yellow stars. (c) Chemical structures of the substrate and substrate analog used in this study.

In this study, we aim to identify regions of the triple mutant myoglobin that exhibit altered HDX behavior compared to its WT′ counterpart. The initial step in the HDX‐MS experiment involved generating a comprehensive peptide library through endopeptidase digestion. Each of the expressed protein has a total of 162 amino acids including the six‐histidine affinity tag at the C‐terminus of the sequence. Both myoglobin and its triple mutant produced around 30 high‐quality peptides from proteolytic cleavage, among which 9 reproducible and non‐overlapping peptides were selected for detailed analysis, while the remaining peptides were analyzed as supporting data to confirm consistency across overlapping regions. Sequences of these 9 non‐overlapping peptides and other overlapping peptides are provided in Table S2. These peptides make up 97% of the protein sequence (Figure 2), providing an opportunity to view the holistic and spatial resolution of HDX results on the protein structure.

### 2.2 Time‐resolved HDX experiment unveiled HDX behavior change in the triple mutant in apo state

**FIGURE 3:**
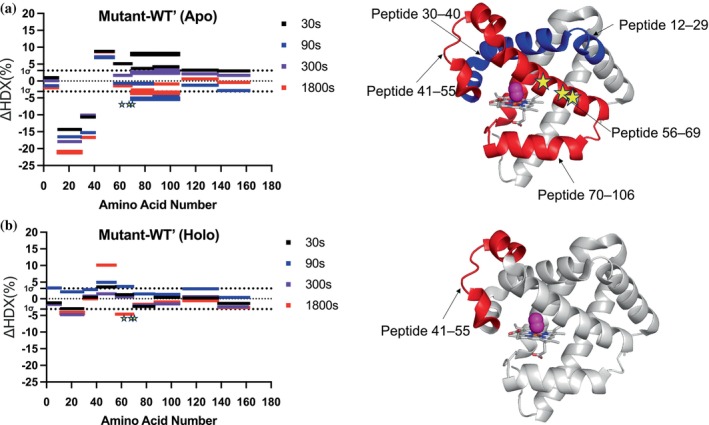
Comparison of HDX behavior between WT′ myoglobin and the engineered triple mutant using Wood's plots. Changes in deuterium uptake (ΔHDX%) across amino acid residues are shown for the apo state (a) and substrate analog‐bound state (b) at four time points: 30 s (black), 90 s (blue), 300 s (purple), and 1800 s (red). Data at 3600 and 5400 s were collected but are not shown in the plot, as exchange was largely saturated and yielded no further mechanistic insight. The dotted horizontal lines at 1σ (3.1%) ΔHDX reflects the experimental variability of the HDX workflow and was used as the cutoff for identifying significant uptake differences. ΔHDX represent the confidence interval, indicating the threshold for significant differences in hydrogen‐deuterium exchange. Bars extending beyond these boundaries reflect meaningful alterations in protein dynamics. The x‐axis represents amino acid number, while the y‐axis shows the percentage change in HDX uptake. In the apo state, notable differences are observed for peptides 12–29, 30–40, 41–55, 56–69, and 70–106. In the substrate‐bound state, the only peptide showing significant differences is 41–55. Mutation sites are indicated with yellow stars.

We first conducted time‐resolved HDX‐MS experiments at 25°C for both WT′ myoglobin and its triple mutant in the apo state, measuring deuterium uptake at seven time points (0, 30, 90, 300, 1800, 3600, and 5400 s). The MS data confirm that the HDX process studied here follows apparent EX‐2 kinetics (representative spectra demonstrating EX2 behavior are shown in Figure S2) in all cases, reflecting local and reversible protein unfolding where *k*_close_ ≫ *k*_int_. Chromatographic retention times for each of the peptides were constant throughout the LC run. The extent of deuterium incorporation at each time point was quantified by calculating the mass difference between deuterated and non‐deuterated samples using HDX Workbench (Pascal et al. 2012). Subsequently, HDX uptake curves were generated as a function of time for all non‐overlapping peptides, achieving a 97% sequence coverage (Figure 2b). The results revealed a clear time‐dependent increase in deuterium uptake for most peptides, demonstrating a progressive exchange with increasing labeling duration (Figure S2). To further assess structural and dynamic differences between myoglobin and its triple mutant, we constructed Wood's plots comparing their relative deuterium uptake. For statistical interpretation of HDX differences, we generated a global 1σ threshold by pooling all replicate deuterium uptake values (across all peptides and labeling times) and calculating the standard deviation of this distribution. This pooled variance estimate (1σ = 3.1%) reflects the experimental variability of the HDX workflow and was used as the cutoff for identifying significant uptake differences. As shown in Figure 3a, several peptides exhibited significant differences in deuterium uptake, indicating localized alterations in protein dynamics for the triple mutant.

Peptides showing changes in HDX uptake include peptide 12–29, peptide 30–40, peptide 41–55, peptide 56–69, and peptide 70–106, collectively accounting for more than 50% of the protein sequence (mapped on structure using PDB 6Z4T) (Figure 3a). Among these, peptide 68–71 contains the V64Q/T67I/A68G mutations. This suggests that the observed differences in deuterium uptake arise from both direct local perturbations near the mutation site and propagated long‐range effects that influence the overall conformational dynamics. The observed HDX effects can be broadly categorized into two general types: peptides exhibiting increased flexibility, which show higher deuterium uptake due to structural dynamics changes in the triple mutant, and peptides exhibiting decreased flexibility, which demonstrate lower deuterium uptake. These differences provide insights into localized conformational changes and alterations in protein dynamics. Peptide 12–29 exhibits the most pronounced decrease in HDX uptake at all timepoints, with an average reduction of 18%, followed by peptide 30–40, which shows a 13% decrease in average. In contrast, regions exhibiting greater flexibility display relatively lower HDX changes (peptides 41–55, 56–69, and 70–106), with an average reduction below 10%, compared to more rigidified peptides. These findings suggest that the evolved dynamics in the triple mutant in its native state achieve a balanced interplay between flexibility and rigidity, where stabilized regions exhibit a moderated reduction in deuterium exchange, while more dynamic segments retain the ability to undergo controlled hydrogen‐deuterium exchange. This equilibrium may contribute to an optimized conformational landscape, allowing the triple mutant to exhibit evolved dynamics that are beneficial for its function (Figure S2).

### 2.3 HDX analysis shows minimal difference in myoglobin mutant in the presence of the analog

To investigate how protein dynamics evolve in the presence of the ligand for the mutant, we first synthesized a modified analog by replacing the B‐ring phenol group of the substrate with a cyclohexyl group (Figure 2c). This modification prevents undesired reaction with the protein during HDX experiments while preserving structural resemblance to uncover dynamic effects. HDX experiments with saturated analog (99.0% complex formation) were performed under the same conditions as those used for the apo‐state studies to ensure direct comparability of deuterium exchange patterns. HDX uptake plots for both myoglobin and its triple mutant in the presence of the substrate analog were generated as a function of time for all non‐overlapping peptides (Figure S2). We also constructed Wood's plots to compare the HDX uptake of the triple mutant with that of the WT′ myoglobin, allowing for a clear visualization of differences in deuterium exchange patterns using the same confidence interval. However, only one major peptide, peptide 41–55, exhibits a significant difference in HDX uptake at the longest timepoint (Figure 3b). This peptide adopts a helix–loop–helix motif and follows peptide 56–69, which contains the mutation sites, suggesting a potential structural and dynamic coupling between these regions, even though the changes in dynamics for peptide 56–69 are masked upon ligand binding. This observation contrasts with the apo‐state studies, where five peptides exhibited significant changes in HDX uptake, suggesting that in the apo state, the protein in its native conformation displays broader dynamic behavior. Studies have shown that, in most cases, ligand binding naturally restricts protein dynamics, leading to a more conformationally stable state (Gao et al. 2020). This reduction in dynamics is often attributed to ligand‐induced structural stabilization, where binding interactions reinforce specific conformations and limit local fluctuations. Ligand binding frequently enhances hydrogen bonding networks, reduces backbone mobility, and decreases overall structural fluctuations, resulting in lower HDX uptake. This phenomenon is widely observed in allosteric regulation, enzyme–substrate interactions, and protein–ligand complexes, where intrinsically dynamic regions become more ordered upon binding (Gao et al. 2022).

### 2.4 HDX reveals differences in binding effects between WT′ and mutant myoglobin

**FIGURE 4:**
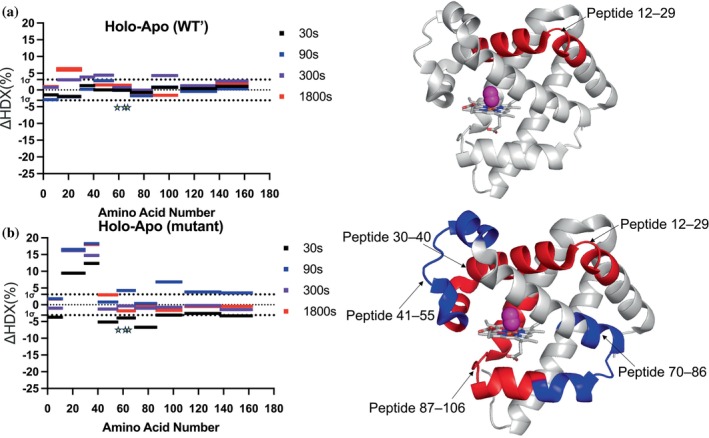
Comparison of ligand‐induced HDX changes in WT′ and mutant myoglobin using Wood's plots. Changes in deuterium uptake (ΔHDX%) across amino acid residues are shown between the ligand‐bound and apo states at four time points: 30 s (black), 90 s (blue), 300 s (purple), and 1800 s (red). Data at 3600 and 5400 s were collected but are not shown in the plot, as exchange was largely saturated and yielded no further mechanistic insight. The dotted horizontal lines at 1σ (3.1%) ΔHDX mark the confidence interval, with bars extending beyond these thresholds indicating significant alterations in hydrogen‐deuterium exchange. The x‐axis represents amino acid number, while the y‐axis denotes the percentage change in HDX uptake. For WT′ myoglobin, notable differences are observed only in peptide 12–29, whereas in the mutant, significant changes are detected in peptides 12–29, 30–40, 41–55, 70–86, and 87–106. Mutation sites are indicated with yellow stars.

To gain deeper insight into how the binding of the substrate analog influences the structural dynamics and protein motions, we compared the HDX uptake results for both the WT′ and triple‐mutant myoglobin between their apo and holo states. To maintain ≥99% complex formation during HDX labeling, we used the substrate analog at a more than tenfold molar excess over the protein, ensuring that virtually all protein molecules remained bound throughout the experiment. This excess concentration ensures near‐complete saturation of the protein with the analog, effectively minimizing the presence of the unbound state throughout the entire experimental time window. We then constructed Wood's plots for both proteins. As shown in Figure 4a, the only peptide that shows significant HDX change upon binding for the wild type of myoglobin is peptide 12–29.

**FIGURE 5:**
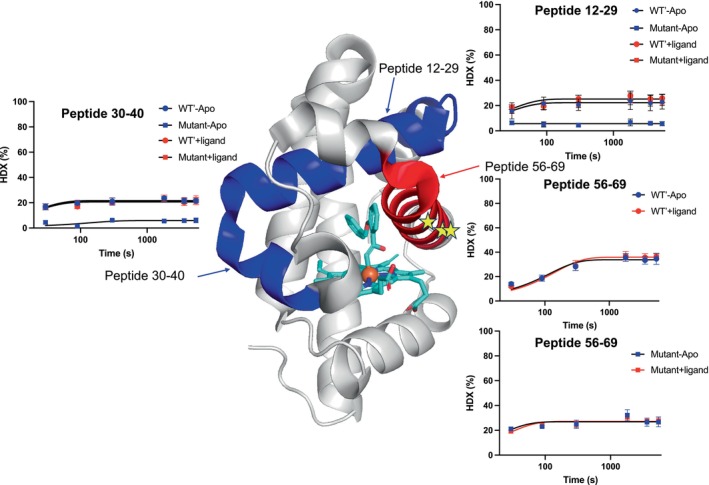
Structural regions showing altered HDX behavior upon ligand binding or mutation. Peptides (12–29, 30–40, and 56–69) showing different HDX behaviors due to binding of ligand or mutation are colored in red (more flexible) and blue (more rigid). These peptides correspond to N‐terminus helixes of the structure. Deuterium uptake plots for these three peptides are shown [apo‐WT′ (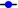), WT′ with ligand (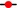), apo‐triple mutant (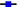), and triple mutant with ligand (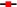)]. For peptide 56–69, the HDX plots are divided into two plots to show the HDX pattern change upon mutation. Mutation sites are indicated with yellow stars.

Most studies report that protein dynamics are generally restricted or “locked down” upon ligand or analog binding, leading to reduced flexibility and solvent exposure. This is typically attributed to the stabilization of the protein's conformational ensemble through increased intramolecular interactions or structural rigidification. However, in the case of myoglobin, our HDX analysis reveals a slight increase (~6%) in deuterium uptake upon substrate analog binding. This observation suggests that, rather than restricting motion, the binding event induces a more dynamic conformational state, potentially due to subtle rearrangements in secondary structural elements or enhanced local breathing motions. Such an increase in flexibility may reflect an adaptive mechanism that facilitates the unnatural ligand accommodation within the myoglobin fold. To evaluate how this binding effect compares to the evolved triple mutant, we analyzed its HDX data afterwards. As illustrated in the Wood's plots (Figure 4b), the HDX changes observed in the mutant are significantly more pronounced, indicating a more substantial impact on protein dynamics. This suggests that the mutations have altered the structural dynamics, potentially enhancing its responsiveness to ligand binding. We identified a total of five peptides exhibiting HDX differences upon ligand binding: peptide 12–29, peptide 30–40, peptide 41–55, peptide 70–86, and peptide 98–108. The impact is twofold: some peptides exhibit increased HDX exchange, suggesting enhanced flexibility or greater solvent accessibility, while others show decreased HDX exchange, indicating regions of reduced conformational dynamics. This differential effect suggests that the mutations may induce global changes in protein motion, potentially altering the overall dynamic landscape of myoglobin in response to ligand binding. This observation aligns with the mutational impacts observed in the apo state (Figure 3a), though the direction of the HDX changes is notably reversed. In the apo state, regions that exhibited increased HDX exchange upon mutation now show decreased exchange upon binding of ligand for the mutant, and vice versa. This inversion suggests that the substrate analog binding may differentially modulate the structural dynamics of the WT′ and mutant myoglobin, potentially altering their conformational flexibility in distinct ways. Despite these differences in effect, the same regions of the protein are consistently affected across both HDX comparative studies. However, the remaining helical structures, located at the back of the protein based on the orientation in Figures 4 and 5, remain largely unaltered in all HDX studies, indicating their structural stability despite ligand binding or mutation. This pattern underscores the presence of key structural elements that are particularly responsive to both mutation and ligand binding, suggesting that these regions play a pivotal role in protein function. Their pronounced sensitivity to structural perturbations and substrate interactions strongly indicates their role in shaping the protein's conformational landscape for functional dynamics.

### 2.5 The myoglobin triple mutant in complex with a ligand exhibits altered HDX kinetics compared to its WT′ counterpart

While Wood's plots are highly informative for comparing HDX uptake differences between conditions, they do not provide insights into the underlying HDX kinetics for peptides with HDX changes. Mapping HDX changes on the protein structure effectively highlights spatial differences in deuterium incorporation but lack the ability to reveal exchange rate trends or subtle kinetic shifts across time points. To achieve a more comprehensive understanding of how substrate binding and mutation influence HDX uptake thermodynamics and kinetics, further analysis is required through HDX uptake pattern visualization and data fitting approaches. By fitting time‐resolved HDX data, we can better characterize region‐specific exchange rates, differentiate between fast and slow‐exchanging regions (Gao et al. 2020), and assess how ligand binding or mutation influences HDX exchange rates.

To perform such analysis, all the HDX data were fitted to one exponential decay model using GraphPad Prism 10 (Swift 1997) and fitted parameters can be found in Table S3. Pattern inspection reveals binding and mutational effects. As shown in Figure 5, out of all the analyzed peptides, three peptides (peptides 12–29, 30–40, and 56–69) show significant differences in deuteron uptake. These results overlap with the Wood's plots analyses. The remaining peptides did not show visible perturbation either by binding of ligand or mutation. The secondary structure of myoglobin is predominantly α‐helical. There are eight alpha helices, designated as A through H (Helix A [residues 3–18]; Helix B [residues 20–35]; Helix C [residues 36–42]; Helix D [residues 49–55]; Helix E [residues 58–77]; Helix F [residues 86–95]; Helix G [residues 100–118]; and Helix H [residues 120–147]). These helices are connected by loops and turns. Peptide 12–29 is the first two helices that reside on top of the bound substrate, whereas peptide 30–40 is turned towards the heme cofactor. Peptide 56–69 is in close proximity to the ring structure of the substrate, harboring the mutation sites of V64Q/T67I/A68G.

For peptide 12–29, the WT′‐apo, WT′‐ligand, and triple mutant‐ligand exhibited highly similar HDX exchange patterns, with the only deviation arising in the apo state of the triple mutant. This finding indicates that the mutations constrain local conformational dynamics in the absence of ligand. Remarkably, ligand binding restores the exchange behavior to that of the wild type. This observation mirrors the result from Wood's plots. This is not an isolated case; peptide 30–40 showed the same pattern as peptide 12–29. This repeated pattern seems to resonate with the innate dynamic nature of proteins. This property of constant flux produces a wide range of distributed substates referred to as the conformational landscape, which is treated as a continuum of thermodynamic states at equilibrium. Fluctuations within a protein permit jump between ensembles over relatively low energy barriers and lead to rapid sampling of heterogeneous landscapes. Transitions between distinct conformations on different timescales can be coupled to facilitate enzyme catalysis. In the case of the myoglobin, a more rigid conformational landscape limits the enzyme's sampling of productive conformations. By stabilizing conformations that are conducive to catalysis, the active site is properly configured for substrate binding and catalysis, enhancing the enzyme's efficiency.

For peptide 56–69, the behavior is distinctive from peptides 12–29 and 30–40. When fitting the wild type of myoglobin data to a one‐exponential equation, the observed *k*_HDX_ for apo and ligand bound was 0.0052 s^−1^ (0.31 min^−1^) and 0.0046 (0.27 min^−1^), which matched the reported intermediate‐exchanging amides rate (Table S3). The overlapped HDX plots indicate that the binding of the ligand did not affect either the total HDX percentage or the HDX exchange rate. In an intriguing contrast, the HDX curves for the triple mutant are distinct. Exponential fittings show much faster rates for both the apo, and ligand bound states: 0.0447 s^−1^ (2.68 min^−1^) and 0.0263 s^−1^ (1.58 min^−1^), respectively (Table S3). HDX plots for apo and ligand bound states are superimposable. This observed phenomenon reveals a more dynamic and flexible structure for the mutant in this region. However, we were unable to observe the same HDX kinetic change for peptides 12–29 and 30–40, as there was no obvious difference in *k*_HDX_ in any of the comparisons. Enzymatic reactions often require precise positioning of catalytic residues. The increased flexibility in this helical region may enable the active site to adopt active conformations more frequently to optimally position active site residues for iron–carbene intermediate formation which is the rate determining step of the reaction.

The mutational effects observed through HDX rate constants complement Wood's plot analyses by revealing HDX kinetic behaviors that may otherwise be overlooked, thereby providing a more comprehensive understanding of conformational dynamics. This compensatory flexibility adjustment observed between the two sets of peptides is hypothesized to fine‐tune the balance between structural flexibility and rigidity, thereby facilitating the evolution of a more catalytically efficient enzyme for the cyclization reaction. This phenomenon aligns with the Goldilocks Principle, which posits that enzymatic activity is optimized when structural dynamics are finely tuned—neither excessively flexible nor overly rigid (Dorantes‐Gilardi et al. 2018; Gheeraert et al. 2023). Achieving this balance is critical for enzymatic efficiency, as excessive flexibility can lead to structural destabilization, whereas excessive rigidity may hinder the conformational transitions necessary for catalysis. This principle is well established in the field of protein dynamics, where extensive studies have demonstrated that finely regulated structural adaptability is essential for substrate recognition, transition‐state stabilization, and overall enzymatic function.

### 2.6 Molecular basis for the evolution of dynamics in the triple mutant and its role in accelerated catalysis

**FIGURE 6:**
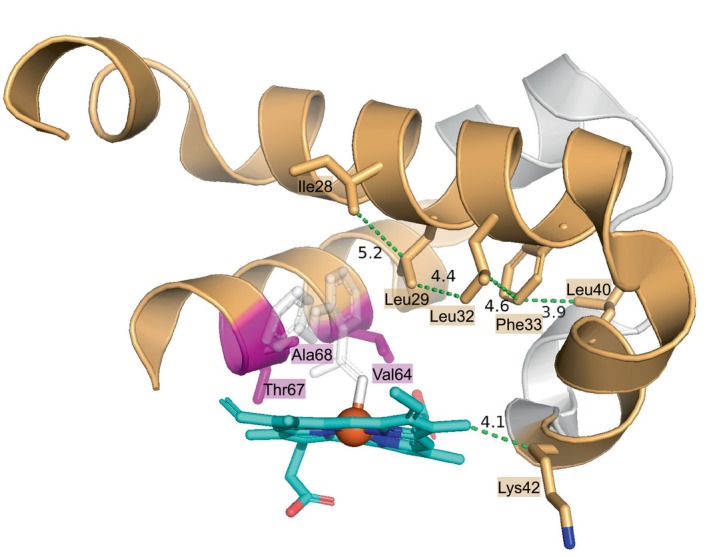
Detailed interactions between peptides 12–29, 30–40, and 56–69 (wheat‐colored) and the bound cofactor. Interactions are depicted as green dashed lines, with distances labeled in Å. Residues Ile28, Leu29, Leu32, Phe33, Leu40, and Lys42 are shown as sticks. Mutation sites of the triple mutant in this study (Val64, Thr67, and Ala68) are highlighted in magenta sticks. The heme cofactor is represented in cyan sticks, with the iron atom colored orange. The docked substrate is depicted in white with partial transparency for clarity.

To gain insights into the molecular mechanisms by which the evolved structural dynamics of the triple mutant enhance the cyclization reaction, we conducted a detailed examination of the molecular interactions between the peptides (12–29, 30–40, 56–69) and the protein structure. As shown in Figure 6, peptides 12–29 and 30–40 harbor a hydrophobic patch, composed of Ile28, Leu29, Leu32, Phe33, and Leu40, that surrounds the heme cofactor and potential substrate. All these hydrophobic residues are within Van der Waals distance of each other (see dashed line in Figure 6 for labeled distances) and oriented towards the active site, creating a properly packed environment. Based on the positioning of residue Ile28 and Leu29, they could form hydrophobic interactions with the phenol group of the substrate for stabilization. In addition, the carbon atom of the side chain of Lys42 is in Van der Waals distance (4.1 Å) from the heme cofactor. For peptide 56–69, potential hydrophobic interactions between the phenol ring of the substrate and Val64, Ala68, and Leu69 could also reinforce the binding. In the triple mutant (V64Q/T67I/A68G), two of the three stabilizing hydrophobic interactions (Val64 and Ala68) are perturbed, causing the helix structure to be loosened up and more flexible. The third mutation T67 is oriented towards the solvent without direct contact with the substrate and cofactor. Changing threonine to a bulkier side chain can influence the overall dynamics of the helical structure by tuning the solvent accessibility and hydrogen bonding. The hydroxyl group of the original Thr residue in position 67 forms two hydrogen bonds with a nearby water molecule and the backbone oxygen of Lys63 (Figure S3). Upon mutation to the Ile residue, this position becomes less protected which results in faster HDX exchange as demonstrated in the HDX results.

In the triple mutant (V64Q/T67I/A68G), the position 64 originally was occupied by a histidine in the WT myoglobin. Typically, this histidine residue serves as the axial ligand to the iron center, influencing the electronic environment of the Fe(II) and Fe(IV) states prior to and after substrate binding. Introducing a histidine‐to‐valine mutation was the first step in conferring carbene transfer activity to myoglobin. However, in the triple mutant, this has been replaced by a glutamine. The lower pKa of glutamine is advantageous for the reaction, as it can facilitate the release of nitrogen gas (N_2_) from the diazo compound by influencing proton transfers, a key step in generating the carbene. In addition, the amide group in glutamine can potentially participate in hydrogen bonding and stabilize the intermediates or transition states involved in carbene formation. The hydrophobic residues in positions 67 and 68 provide a rearranged and optimized hydrophobic environment to improve the positioning of the diazo compound for efficient N_2_ release and carbene generation. This explains the observed HDX behavior for peptide 56–69 that shows a distinct dynamic nature in the triple mutant, allowing for more effective sampling of productive conformations. Collectively, this promotes the rate‐limiting step of carbene formation that leads to a more active protein variant for the intramolecular cyclization reaction.

### 2.7 Computational studies validate HDX findings on protein dynamics and its role in catalysis for the triple‐mutant myoglobin

To further support the findings from HDX, we also employed computational approaches to validate our results. Computational studies provide molecular‐level insights into protein dynamics, allowing us to analyze conformational flexibility, structural stability, and key interactions that may contribute to catalysis. By integrating both experimental and computational methods, we aim to obtain a more comprehensive understanding of how the triple‐mutant myoglobin's structural dynamics influence its enzymatic function.

To elucidate the binding interactions between WT′ and mutant myoglobin with its substrate, we employed protein modeling and molecular docking experiments. The three‐dimensional structure of the mutant myoglobin was predicted using the Rosetta software suite (Leman et al. 2020), renowned for its robust capabilities in protein structure prediction and design. Subsequent to structural modeling, we performed molecular docking studies utilizing AutoDock (Morris et al. 2008), a widely recognized tool for predicting ligand binding to macromolecules. AutoDock employs algorithms such as the Lamarckian Genetic Algorithm coupled with an empirical free energy scoring function to predict optimal binding conformations and affinities. Prior to docking, both WT′ and mutant myoglobin structures underwent rigorous preparation protocols to rectify any structural inconsistencies and to optimize their geometries, ensuring the reliability of the simulations. The docking results revealed notable differences in binding energies between the WT′ and mutant myoglobin. The WT′ myoglobin exhibited a binding energy of −9.74 kcal/mol, whereas the triple mutant variant demonstrated a more negative binding energy of −11.82 kcal/mol, indicative of enhanced ligand binding affinity in the mutant (see Table 1). The dominant contribution to the binding energy difference arises from van der Waals (vdW) interactions. These findings suggest that the introduced mutations in myoglobin may lead to conformational changes that favor stronger ligand interactions.

**TABLE 1:** Calculated binding energy (unit: kcal/mol) for wild‐type myoglobin and the triple mutant with the substrate.

Ligand–protein complex	ΔG_binding (kcal/mol)	ΔE_elec (kcal/mol)	ΔE_vdW (kcal/mol)	ΔG_polar (kcal/mol)	ΔG_nonpolar (kcal/mol)
WT′–ligand	−9.74	0.17	−14.69	4.78	−14.69
Triple mutant–ligand	−11.82	0.14	−16.73	4.77	−16.73

**FIGURE 7:**
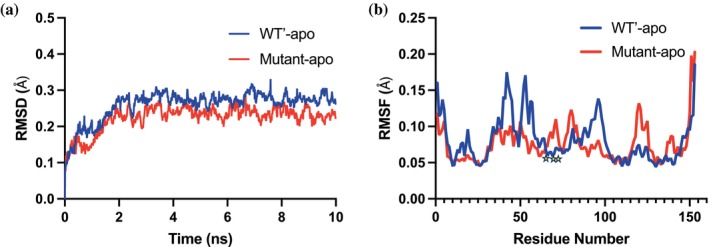
Molecular dynamics (MD) simulation results for WT′ myoglobin (blue) and the triple mutant (red). (a) Root‐mean‐square deviation (RMSD) as a function of time, showing structural deviations from the initial conformation. (b) Root‐mean‐square fluctuation (RMSF) as a function of residue number, indicating the flexibility of individual residues. Mutation sites are indicated with yellow stars.

To further elucidate the impact of the triple mutation on myoglobin's structural dynamics, we conducted a 10‐nanosecond molecular dynamics (MD) simulation using GROMACS (Pronk et al. 2013) for both apo WT′ and triple mutant proteins and analyzed the root mean square deviation (RMSD) for both protein systems and the root mean square fluctuation (RMSF) for each amino acid. RMSD quantifies structural deviations over time, providing insights into protein flexibility and stability. A low and stable RMSD suggests minimal conformational changes indicative of a stable system, whereas an increasing or fluctuating RMSD reflects flexibility, domain movements, or ligand‐induced conformational shifts. The RMSF further characterizes the flexibility of individual amino acid residues by measuring their average positional deviations over the simulation period. Our comparative analysis revealed that during the MD simulations, the triple mutant exhibited a lower RMSD, indicating a more stable system relative to WT′ myoglobin (Figure 7a).

To examine residue‐level fluctuations in detail, we analyzed the RMSF data for both simulations. As shown in Figure 7b, we identified four major regions with distinct flexibility differences between the two proteins, specifically amino acids 40–43, 52–57, 95–98, and 119–121. Among these, the most pronounced changes were observed in the first two regions (amino acids 40–43 and 52–57), both of which exhibited reduced fluctuations. When compared with HDX analyses, we found that these two regions correspond to peptides 30–40 and 41–55, which also displayed significant HDX differences (Figure 3). Notably, while amino acids 40–43 demonstrated decreased flexibility in both HDX analyses and MD simulations, amino acids 52–57 exhibited opposing flexibility trends between the two methods. This discrepancy is expected, as MD simulations capture nanosecond fluctuations, whereas HDX experiments reflect millisecond‐to‐hour‐scale dynamics. Despite these differences in timescale, both approaches consistently identified regions undergoing dynamic changes within the protein, suggesting the presence of dynamic coupling, where local, fast‐timescale fluctuations observed in MD propagate through the protein and influence larger, slower conformational transitions detectable by HDX. Such interconnected motion networks are fundamental to protein function, facilitating allosteric regulation, structural adaptability, and catalytic efficiency. These findings reinforce the notion that protein dynamics are not merely independent fluctuations but rather hierarchical and coordinated processes that contribute to functional adaptability (Henzler‐Wildman et al. 2007).

## 3 DISCUSSION

Despite significant advances in understanding the fundamental aspects of enzyme conformational landscapes and their relationship to catalysis and evolution, the practical application of this knowledge in enzyme engineering remains limited (Arnold 2018; Bornscheuer et al. 2012; Chin 2017; Tokuriki and Tawfik 2009; Yang and Arnold 2021; Zetzsche and Narayan 2020). Research has demonstrated that laboratory‐directed evolution is a powerful strategy to incrementally modify the conformational ensemble of protein scaffolds, narrowing the ensemble to favor more catalytically active states and thereby enhancing enzymatic transformations (Karamitros et al. 2022; Surpeta et al. 2020; Vidal et al. 2023). The mutations accumulated through directed evolution induce global and local conformational changes, which cooperatively organize catalytic residues (Broom et al. 2020; Campbell et al. 2016; Campbell et al. 2018). Thus, the development of protein catalysts for diverse chemical transformations could be significantly advanced by promoting the sampling of productive conformational substates through mutation design during the engineering process (Crean et al. 2020; Nam and Wolf‐Watz 2023; Otten et al. 2020; Schenkmayerova et al. 2021).

In this study, we use myoglobin and its engineered mutant (V64Q/T67I/A68G) as a model system to investigate the relationship between protein conformational dynamics and enzymatic function (Gao et al. 2025). We conducted time‐resolved HDX experiments for both proteins in the presence and absence of the substrate analog. HDX is a powerful biophysical technique for probing protein dynamics with spatial resolution and has been widely applied in studying protein‐ligand interactions, protein–protein interactions, and allosteric mechanisms in cellular processes. Detailed HDX analysis of the apo state for both proteins identified major peptides (peptide 12–29, peptide 30–40, peptide 41–55, peptide 56–69, and peptide 70–106) that exhibit differential behavior in the evolved triple mutant, encompassing both local and distal regions from the mutation sites. These impacted peptides displayed two distinct behaviors, either an increase or decrease in HDX uptake, suggesting modulated structural dynamics that enhance catalysis. In the triple mutant, the heme iron ligand was mutated to glutamine, which has a lower pKa, facilitating nitrogen (N_2_) release from the diazo compound by influencing proton transfers—a key step in carbene generation. Additionally, glutamine can form hydrogen bonding interactions with the reactant, stabilizing the carbene intermediates or transition states involved in its formation. The tuned hydrophobic environment further enhances the positioning of the diazo compound for efficient N_2_ release and carbene formation, contributing to the enzyme's improved catalytic efficiency.

Extending the HDX analyses to the substrate analog‐bound state revealed fewer changes in HDX uptake, with only one peptide (peptide 41–55) showing a marginal difference, likely due to the stabilizing effect of ligand binding, which restricts conformational dynamics. To further explore the binding effect of the analog on both WT′ and mutant myoglobin, we compared HDX uptake differences between the apo and holo states for both proteins. We observed differential binding impacts between the two proteins. Although only one peptide (peptide 12–29) exhibited differences in HDX uptake upon ligand binding in WT′ myoglobin, a similar set of peptides displayed ligand‐binding effects as well as mutational effects in the apo state. This observation suggests a direct link between evolutionary mutations and substrate recognition dynamics, highlighting the interplay between structural evolution and functional adaptation. In addition, analysis of HDX rate constants identified one major peptide (peptide 56–69) exhibiting distinct kinetic behaviors between WT′ and mutated myoglobin—an insight that would not have been possible through Wood's plot analysis alone. Collectively, HDX has identified regions of myoglobin that contribute to its enhanced catalytic performance via dynamic evolution. Our molecular dynamics (MD) simulations further support these findings by highlighting residues with altered fluctuations and demonstrating a more stable overall system, as evidenced by reduced root mean square deviation (RMSD) in the mutant compared to WT′ myoglobin.

In summary, the enhanced catalytic efficiency of the triple mutant is attributed to fine‐tuned conformational dynamics and favorable binding interactions. Mutation‐induced stabilization of protein regions and localized dynamics optimize the enzyme's active site geometry, promoting more precise substrate recognition and catalysis. These findings underscore the interplay between structural stability and dynamic flexibility in governing enzymatic function, reinforcing the role of conformational dynamics in enzyme evolution and engineering.

## 4 CONCLUSIONS

In this work, we established myoglobin and its engineered triple mutant (V64Q/T67I/A68G) as a model system to dissect the relationship between conformational dynamics and catalytic function. Using time‐resolved HDX‐MS, we identified distinct regions across the protein scaffold where mutation‐induced changes in flexibility contribute to catalytic efficiency. These results demonstrate that mutations not only locally alter dynamics near the active site but also propagate long‐range effects that reshape distal structural elements, thereby fine‐tuning the overall conformational ensemble to favor catalytically competent states. Analysis of both apo and ligand‐bound forms revealed that the evolutionary mutations directly influence substrate recognition dynamics, linking structural evolution to functional adaptation. Notably, ligand binding in the mutant system stabilized conformational fluctuations in key regions, reflecting an optimized interplay between structural rigidity and dynamic flexibility. Complementary MD simulations further confirmed that the mutant exhibits a globally more stable conformational landscape, while maintaining sufficient flexibility to support substrate engagement and turnover. Together, these findings highlight how subtle conformational reprogramming, rather than large‐scale structural rearrangements, underpins the emergence of enhanced catalytic activities.

Overall, this study underscores the critical role of conformational dynamics in governing enzyme catalysis, providing both mechanistic understanding and design principles for future engineering efforts. By integrating HDX‐MS with computational approaches, our framework offers a generalizable strategy to map mutation‐induced dynamic effects and link them to function. These insights extend beyond myoglobin, with broader implications for the rational design and directed evolution of enzymes for biocatalysis, synthetic chemistry, and biotechnology.

## 5 MATERIALS AND METHODS

### 5.1 Reagents

All chemicals and reagents were obtained from commercial suppliers with the highest level of purity. The plasmids harboring the wild type of myoglobin and triple mutant contain an isopropyl β‐D‐1‐thiogalactopyranoside (IPTG) inducible T7 promoter and a kanamycin resistance gene. *E. coli* strains of DH5α and BL21(*DE3*) competent cells were used for plasmid propagation and protein expression, respectively.

### 5.2 Protein expression and purification

The wild type of myoglobin and triple mutant were expressed and purified as stated before (Gao et al. 2025). The purity of the protein was assessed by SDS‐PAGE and intact mass spectrometry (MS). Protein concentration was determined using the Thermo NanoDrop 2000 at 280 nm. The concentration values obtained from NanoDrop were calibrated using the extinction coefficient calculated online (https://web.expasy.org/protparam/).

### 5.3 HDX experiment

Before HDX analysis, a peptide identification run was performed under non‐deuterated conditions to generate a complete peptide library for HDX‐Workbench. This library, which includes all detectable and overlapping peptides produced by proteolysis, is required by the software to assign and quantify deuterium uptake across the full‐time course. To achieve higher than 99.0% ligand binding, excess ground state analog (micromolar binding) was incubated with enzyme to achieve more than 99.0% of bound complex in the course of the HDX experiments. For apo‐state HDX, purified myoglobin was prepared at 100 μM. To initiate deuterium labeling, 5 μL of this protein solution was diluted into 45 μL of labeling buffer (47.7 mM Na_2_HPO_4_, 22 mM KH_2_PO_4_, 8.6 mM NaCl, 2 mM MgSO_4_, 99% D_2_O, pD 7.4) at 25°C. At each labeling time point, the exchange reaction was quenched by adding 50 μL of ice‐cold quench buffer (4M guanidine hydrochloride, 200 mM citric acid, 500 mM TCEP, pH 1.8 in H_2_O), and the sample was immediately placed on ice. Proteolysis was initiated by adding 5 μL of 1 μM pepsin and allowing digestion to proceed for 2 min prior to LC–MS analysis. For ligand‐bound (holo) HDX, the protein and substrate analog were mixed to achieve near‐complete saturation before initiating labeling. Based on our binding titrations, the affinity of the analog for myoglobin is *K*_*D*_ ≈ 11.2 μM. The final concentrations used for HDX were [*P*]_0_ = 100 μM and [*I*]_0_ = 1188 μM, which, when applied to the quadratic binding Equation (1) for a 1:1 complex, yield a fractional occupancy of *θ* ≈ 0.99. Thus, ~99% of the protein molecules,

(1) $$\left[\mathrm{PI}\right]=\frac{\left({\left[P\right]}_{0}+{\left[I\right]}_{0}+K_{D}\right)-\sqrt{\left({\left[P\right]}_{0}+{\left[I\right]}_{0}\right)+{\left(K_{D}\right)}^{2}-4{\left[P\right]}_{0}+{\left[I\right]}_{0}}}{2},$$

were bound to ligand under the labeling conditions. HDX labeling, quenching, and digestion of the holo samples were carried out identically to the apo samples, enabling direct comparison of backbone dynamics between the two states. The digested sample was placed into a Thermo‐Dionex Ultimate 3000 HPLC system autosampler for injection. All HDX experiments were performed in duplicate, with two independent biological replicates to ensure reproducibility.

### 5.4 LC–MS for HDX measurement

For LC–MS/MS analysis, the peptides were separated by a 20 min gradient elution at a flow rate of 115 μL/min with a Thermo‐Dionex Ultimate 3000 HPLC system, which was directly interfaced with a Thermo Scientific Q Exactive mass spectrometer. Peptides were separated on a reverse phase column (Acquity UPLC BEH C18 column 1.7 μm, 2.1*50 mm, Waters, UK). Mobile phase A consisted of 1% formic acid, and mobile phase B consisted of 99% acetonitrile and 1% formic acid. The Q Exactive mass spectrometer was operated in the data‐dependent acquisition mode using Xcalibur 2.0.0.0 software and there was a single full‐scan mass spectrum in the orbitrap (350–2000 m/z, 70,000 resolution). The mass spectrometer was operated at a source temperature of 250°C and a spray voltage of 3.0 kV. Peptic peptides were identified using an in‐house Proteome Discoverer (Version PD1.4, Thermo‐Fisher Scientific, USA). The search criteria were as follows: no enzyme was required; two missed cleavages were allowed; precursor ion mass tolerances were set at 20 ppm for all MS acquired in an orbitrap mass analyzer; and the fragment ion mass tolerance was set at 0.02 Da for all MS spectra acquired. The peptide false discovery rate (FDR) was calculated using Percolator provided by PD. When the *q* value was smaller than 1%, the peptide spectrum match (PSM) was considered to be correct. FDR was determined based on PSMs when searched against the reverse, decoy database. Peptides only assigned to a given protein group were considered unique. The false discovery rate (FDR) was also set to 0.01 for protein identifications. The deuterium exchange levels were determined by subtracting the centroid mass of un‐deuterated peptide from the centroid mass of deuterated peptide using HDX Workbench (Pascal et al. 2012).

### 5.5 Data analysis

The MS data confirm that the HDX process studied here follows apparent EX‐2 kinetics in all cases, reflecting local and reversible protein unfolding where *k*_close_ ≫ *k*_int_. Chromatographic retention times for each of the peptides were constant throughout the LC run. For each of the peptides, the number of incorporated deuterons was obtained by calculating the peptide mass change before and after the exchange process. Mass spectral data acquired for HDX measurements were analyzed using the software, HDX WorkBench. The non‐overlapping 9 peptides from the peptide library were selected as a peptide set (Table S2). Peptide mass spectrometry data were manually curated, focusing on peptide identification, noise, and retention time. The deuteron uptake was calculated for each of the nine peptides in percentage. The data were plotted as deuterons versus time in log scale using prism software. The rates and extents of exchange were determined from one‐exponential fits to the analyzed time‐resolved HDX data. The dataset contains the original HDX data and HDX summary.

### 5.6 Docking experiments and molecular dynamics simulations

The wild type myoglobin was obtained using PDB number 6Z4T and the mutant protein structure was obtained using RoseTTAFold (Baek et al. 2021). Molecular docking simulations were performed using AutoDock 4.2.6 to predict the binding affinity and interaction mode of the ligand with the target protein (Morris et al. 2008). The receptor structure was prepared by removing water molecules and adding polar hydrogen; Kollman charges were assigned to the protein, while ligand partial atomic charges were computed using the AMBER force field within AutoDockTools (Wang et al. 2004). A grid box was centered on the binding site with dimensions 127 × 127 × 127 Å and a spacing of 0.375 Å to encompass the entire active site. The rigid receptor‐flexible ligand approach was applied, allowing full torsional flexibility of the ligand. The Lamarckian genetic algorithm (LGA) was used for conformational sampling, with a population size of 300, a maximum of 2.5 million energy evaluations, and 200 independent docking runs. The binding free energy (ΔG_binding) was estimated from the AutoDock scoring function (Huey et al. 2007) which includes van der Waals, hydrogen bonding, desolvation, electrostatic, and torsional energy components. The best docking pose was selected based on the lowest binding energy and root‐mean‐square deviation (RMSD) clustering criteria.

The MD simulations were performed using the GROMACS Source code package (Lindahl et al. 2001). The OPLS‐AA (Optimized Potential for Liquid Simulations‐All Atom) force field was applied for the proteins (Jorgensen et al. 1996). The protein was solvated in a cubic box with a 10 Å padding of SPC/E (the missing term in effective pair potentials for water molecules), and Na^+^ and Cl^−^ counter ions were added to neutralize the system (Berendsen et al. 1987). Energy minimization was performed using the steepest descent algorithm for 50,000 steps to remove steric clashes. The system was gradually heated to 300 K under the canonical (NVT) ensemble, followed by pressure equilibration under the NPT ensemble using the velocity rescaling (V‐rescale) a modified version of the Berendsen thermostat (Bussi et al. 2007) at 1 bar pressure using a Parrinello‐Rahman barostat (Parrinello and Rahman 1981). The production MD simulation was conducted for 10 ns using the leapfrog integrator with a time step of 2 fs. To ensure statistical robustness, three independent MD simulations were carried out, initiated from distinct starting velocities and/or coordinates. To maintain the stability of hydrogen‐containing bonds, the LINear Constraint Solver (LINCS) algorithm was employed (Hess et al. 1997), and Periodic Boundary Conditions (PBC) were applied in all directions. Electrostatic interactions were treated using the Particle Mesh Ewald (PME) method with a 1.0 nm cutoff for short‐range interactions (Darden et al. 1993).

## AUTHOR CONTRIBUTIONS

**Hanzi Gao:** Writing – original draft; investigation; writing – review and editing; methodology; conceptualization. **Edgar Africano Camargo:** Conceptualization; methodology; investigation; software; data curation; formal analysis; validation. **Jude N. Ubi:** Conceptualization; investigation; methodology. **Xiuyuan Duan:** Investigation; methodology. **Xiaolin Tian:** Methodology; investigation; software; formal analysis. **Haiteng Deng:** Investigation; methodology; software; formal analysis. **Guojun Zheng:** Conceptualization; supervision; project administration; writing – original draft; writing – review and editing; funding acquisition; resources. **Shuaihua Gao:** Conceptualization; investigation; writing – original draft; visualization; validation; methodology; software; data curation; supervision; writing – review and editing.

## CONFLICT OF INTEREST STATEMENT

The authors declare no conflict of interest.

## Supporting information


**Data S1.** Supporting Information figures and tables.

**Data S2.** Original HDX data for WT′ myoglobin and its triple mutant in the absence/presence of ligand.

## Data Availability

The data that support the findings of this study are openly available in PRIDE‐PRoteomics IDEntifications Database at https://www.ebi.ac.uk/pride/, reference number PXD071026.
