# Supplementary material for: Hydrogen‐deuterium exchange reveals catalytically linked protein flexibility in myoglobin‐mediated intramolecular C(sp3)‐H activation
Source: Protein Sci. 2025 Dec 22;35(1):e70410. doi: 10.1002/pro.70410 (PMC12720788; doi:10.1002/pro.70410)
Supplement: Supplementary file 1 — Data S1. Supporting Information figures and tables. [file PRO-35-e70410-s001.docx]

Hydrogen-Deuterium Exchange Reveals Catalytically Linked Protein Flexibility in Myoglobin-Mediated Intramolecular C(sp³)-H Activation

Hanzi Gao^†,^ ^§^, Edgar Africano Camargo^‡,^ ^§^, Jude N Ubi^‡^, Xiuyuan Duan^†^, Xiaolin Tian^⊥^, Haiteng Deng^⊥^, Guojun Zheng^†^*****, Shuaihua Gao^‡^*****.

^†^State Key Laboratory of Chemical Resources Engineering, Beijing University of Chemical Technology, Beijing, 100029, People's Republic of China.

^‡^Department of Chemical and Biomolecular Engineering, Tulane University, New Orleans, Louisiana, 70123, United States.

^⊥^MOE Key Laboratory of Bioinformatics, School of Life Sciences, Tsinghua University, 100084 Beijing, China.

^§^These authors contributed equally to this work.

*To whom correspondence should be addressed, [zhenggj@buct.edu.cn , and](mailto:zhenggj@buct.edu.cn%20,%20and) [sgao11@tulane.edu](mailto:sgao11@tulane.edu).

This PDF file includes:

Figures S1 to S4

Tables S1 to S3

Reference for SI

Legends for Dataset

Other supplementary materials for this manuscript include the following:

Dataset. Original HDX data for WT’ myoglobin and its triple mutant in the absence/presence of ligand.

**Figures and Tables for the HDX-MS Analyses.**


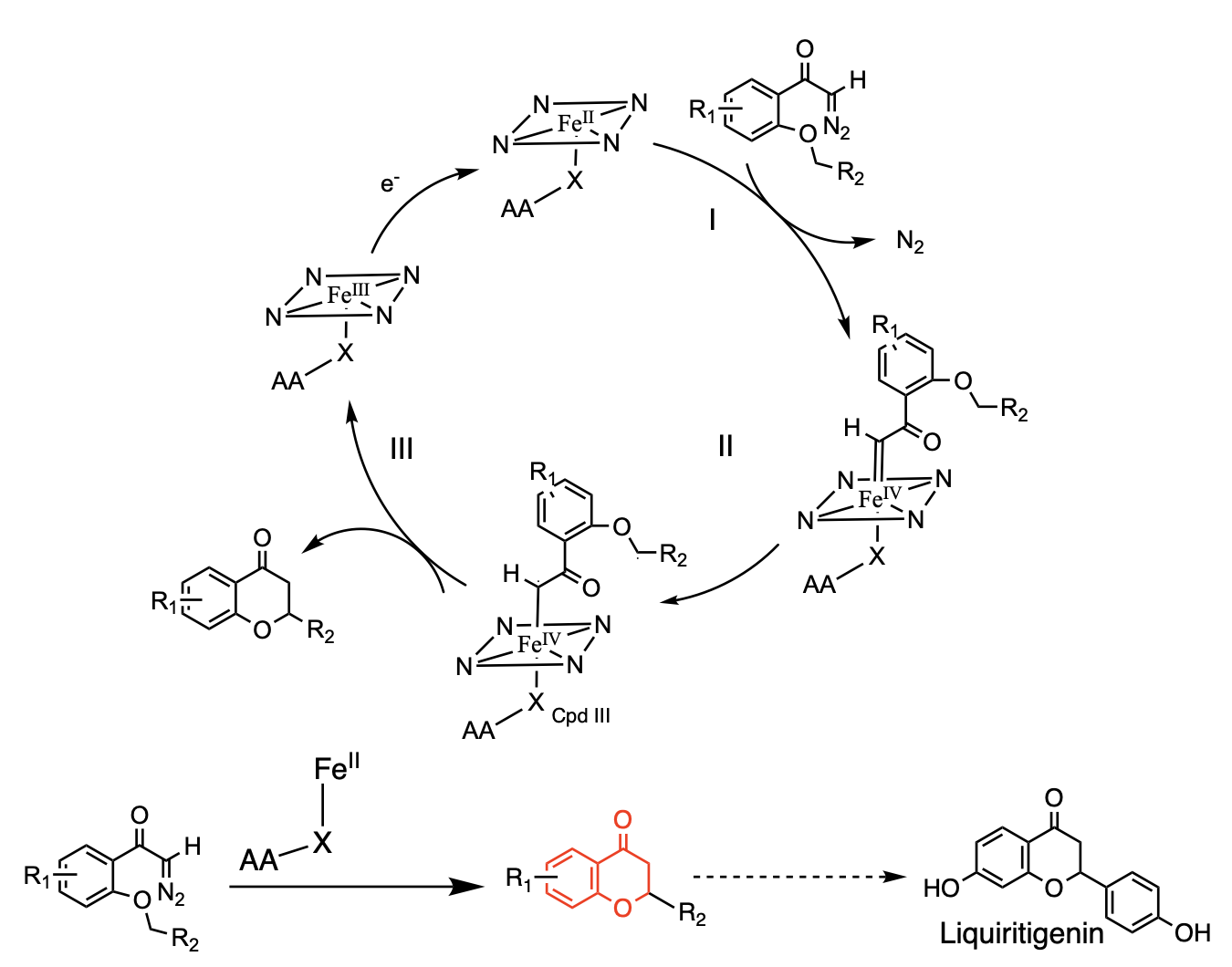


Figure S1. An intramolecular cyclization mechanism utilizing a diazo reagent catalyzed by heme proteins and illustration of the proposed approach for synthesizing pharmacologically active compounds, the anti-inflammatory, antioxidant, and anti-cancer agent liquiritigenin (Gao et al, 2025). The intended reaction mechanism begins with the diazo compound (R2C=N2) being introduced to the active site of the heme protein. The heme protein, in the Fe (II) state after reduction, interacts with the diazo compound. The diazo compound decomposes to release nitrogen gas, generating a reactive carbene species (R2C:) (step I). The carbene species coordinates to the iron center of the heme protein, forming a metal-carbenoid complex (step II). The iron in the heme stabilizes the highly reactive carbene intermediate. This step is crucial as the metal-carbenoid complex is the reactive species that will facilitate the cyclization reaction. The metal-carbenoid complex then undergoes an intramolecular cyclization which leads to the formation of a new ring structure (step III). After the cyclization, the newly formed cyclic product is released from the heme iron center. The heme protein is then regenerated to its original state, ready to catalyze another cyclization reaction with a new diazo molecule. The cyclized product can be used as precursor for preparation of a variety of flavanone drugs such as Liquiritigenin.

**Figure S2. Representative isotopic distribution spectra for selected peptide (56-69) demonstrating EX2 exchange behavior under the experimental conditions. Spectra were collected at multiple labeling time points and show the characteristic binomial-like isotopic envelope shifts consistent with EX2 kinetics.**

Figure S3. HDX plots for (apo WT’ (), WT’ with ligand (), apo triple mutant (), and triple mutant with ligand ()).


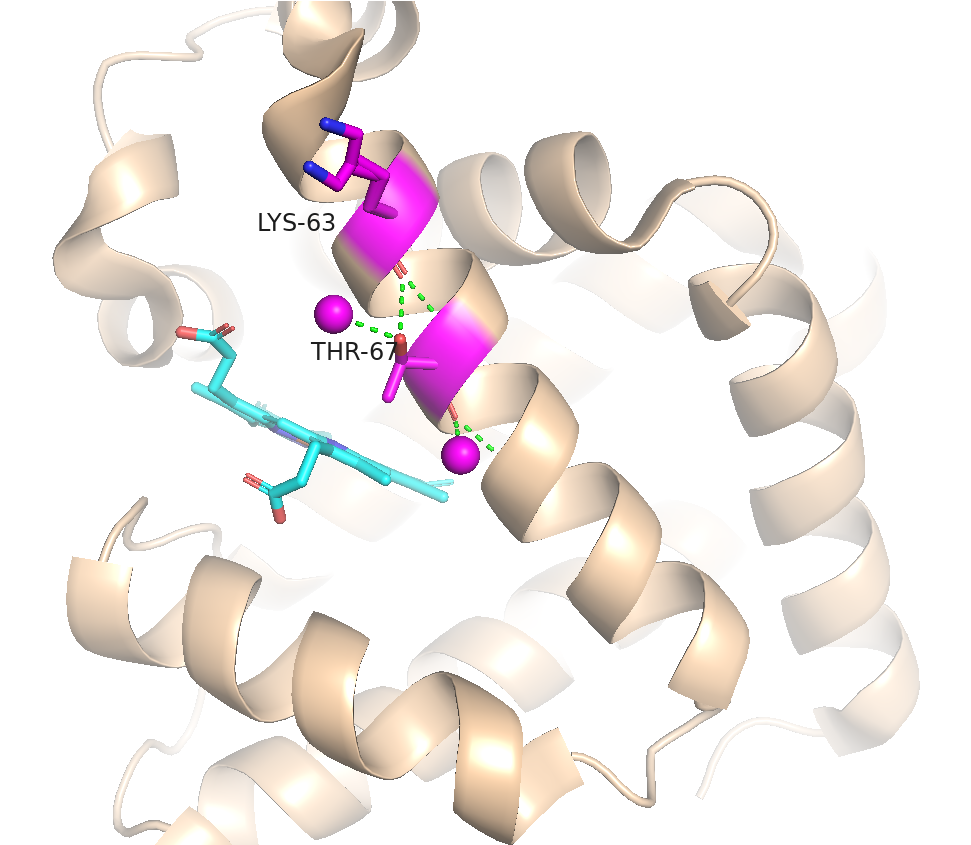


Figure S4. The hydroxyl group of Thr 67 residue forms two hydrogen bonds with a nearby water molecule and backbone oxygen of Lys63. Lys63 in this figure has two conformers as indicated by two sidechain configurations.

Table S1. Catalytic parameters for original myoglobin and its mutant (40). TTN: total turnover number.

| Protein | Yield [%] | ee% (*S*) | TTN |
| --- | --- | --- | --- |
| Wild type | 18.22% | 72.00% | 81 |
| V64Q | 49.38% | 92.20% | 190 |
| V64Q/A68G | 73.00% | 95.70% | 370 |
| V64Q/A68G/T67I | 99.97% | 96.42% | 602 |

Table S2. Overlapping peptide set for myoglobin. The table lists all overlapping pepsin-generated peptides detected in the study. Peptides shown in bold represent the non-overlapping peptide set used in the main analysis and discussed in detail in the manuscript. Peptides containing mutation sites (residues 64, 67, and 68) are annotated in the “Note” column. Triple mutant is this study is V64Q/A68G/T67I.

| Peptide | Length | Amino acid sequence | Note |
| --- | --- | --- | --- |
| Peptide 0–8 | 9 | MVLSEGEWQ |  |
| Peptide 3–7 | 5 | SEGEW |  |
| **Peptide 1–11** | **11** | **VLSEGEWQLVL** |  |
| Peptide 3–11 | 9 | SEGEWQLVL |  |
| **Peptide 12–29** | **18** | **HVWAKVEADVAGHGQDIL** |  |
| Peptide 14–29 | 16 | WAKVEADVAGHGQDIL |  |
| Peptide 20–29 | 10 | ADVAGHGQDI |  |
| **Peptide 30–40** | **11** | **IRLFKSHPETL** |  |
| Peptide 33–54 | 22 | FKSHPETLEKFDRFKHLKTEAEM |  |
| Peptide 33–55 | 23 | FKSHPETLEKFDRFKHLKTEAEMK |  |
| **Peptide 41–55** | **15** | **EKFDRFKHLKTEAEM** |  |
| Peptide 41–69 | 29 | EKFDRFKHLKTEAEMKASEDLKKVGVTAL | Mutation 64, 67, 68 |
| Peptide 42–69 | 28 | KFDRFKHLKTEAEMKASEDLKKVGVTAL | Mutation 64, 67, 68 |
| Peptide 55–69 | 15 | MASEDLKKVGVTAL | Mutation 64, 67, 68 |
| **Peptide 56–69** | **14** | **ASEDLKKVGVTAL** | Mutation 64, 67, 68 |
| Peptide 62–69 | 8 | KVGVTAL | Mutation 64, 67, 68 |
| Peptide 70–76 | 7 | TALGAIL |  |
| **Peptide 70–86** | **17** | **TALGAILKKKGHHEAEL** |  |
| Peptide 77–103 | 27 | KKKGHHEAELKPLAQSHTKHKIPIKY |  |
| **Peptide 70–106** |  | **TALGAILKKKGHHEAELKPLAQSHATKHKIPIKYLEF** |  |
| **Peptide 87–106** | **20** | **KPLAQSHTKHKIPIKYLEF** |  |
| Peptide 110–123 | 14 | AIIHVLHSRHPGDF |  |
| Peptide 110–131 | 22 | AIIHVLHSRHPGDFGADAQGAMNKA |  |
| Peptide 110–134 | 25 | AIIHVLHSRHPGDFGADAQGAMNKALEL |  |
| **Peptide 110–137** | **28** | **AIIHVLHSRHPGDFGADAQGAMNKALELFR** |  |
| Peptide 113–137 | 25 | HVLHSRHPGDFGADAQGAMNKALELFR |  |
| **Peptide 138–162** | **25** | **FRKDIAAKYKELGYQGGSGHHHHHH** |  |
| Average length | 16.52 |  |  |

Table S3. Fitted HDX rate constants for wild type myoglobin and its triple mutant in the absence/presence of ligand. Y= (Y0 - Plateau) *exp(-K*X) + Plateau was used for the exponential fitting. Y0 is the Y value when X (time) is zero. It is expressed in the same units as Y, Plateau is the Y value at infinite times, expressed in the same units as Y. K is the rate constant, expressed in reciprocal of the X axis time units.

| Peptide | Parameter | WT-Apo | Mutant-Apo | WT-Ligand | Mutant-Ligand |
| --- | --- | --- | --- | --- | --- |
| 3-13 | Y0 | 0.06112 | 0.06722 | 0.0662 | 0.06112 |
|  | Plateau | 26.88 | 26.83 | 26.86 | 26.88 |
|  | K | 0.04597 | 0.04118 | 0.05014 | 0.04597 |
| 14-31 | Y0 | -0.0001526 | -4.112e-016 | 0.2324 | -0.0001526 |
|  | Plateau | 22.10 | 5.649 | 24.79 | 22.10 |
|  | K | 0.04198 | Unstable | 0.04101 | 0.04198 |
| 32-42 | Y0 | 0.03866 | 0.0367 | 0.06929 | 0.03866 |
|  | Plateau | 21.54 | 20.858 | 20.40 | 21.54 |
|  | K | 0.04883 | 0.047292 | 0.05040 | 0.04883 |
| 43-57 | Y0 | 0.3753 | 0. 3798 | 0.3077 | 0.3753 |
|  | Plateau | 29.35 | 27.66 | 23.64 | 29.35 |
|  | K | 0.03234 | 0.03657 | 0.03327 | 0.03234 |
| 58-71 | Y0 | 4.966 | 0.2798 | 4.327 | 0.5465 |
|  | Plateau | 33.82 | 26.31 | 35.68 | 27.69 |
|  | K | 0.005239 | 0.004474 | 0.004690 | 0.02633 |
| 72-88 | Y0 | 0.3052 | 0.35230 | 0.4228 | 0.1399 |
|  | Plateau | 50.89 | 50.98 | 51.35 | 49.84 |
|  | K | 0.03960 | 0.03906 | 0.03790 | 0.03905 |
| 89-108 | Y0 | 0.04009 | NA. | 0.1539 | 22.78 |
|  | Plateau | 29.94 | NA. | 30.59 | 30.88 |
|  | K | 0.04603 | NA. | 0.04021 | 0.03147 |
| 72-108 | Y0 | 0.3052 | 0.35230 | 0.4228 | 0.1399 |
|  | Plateau | 50.89 | 50.98 | 51.35 | 49.84 |
|  | K | 0.03960 | 0.03906 | 0.03790 | 0.03905 |
| 112-139 | Y0 | 0.01906 | 0.05094 | 0.05007 | 0.01906 |
|  | Plateau | 25.13 | 25.00 | 24.17 | 25.13 |
|  | K | 0.05146 | 0.05140 | 0.05336 | 0.05146 |
| 140-164 | Y0 | 0.1205 | 0.1700 | 0.2829 | 0.1201 |
|  | Plateau | 24.22 | 25.81 | 26.47 | 24.67 |
|  | K | 0.04037 | 0.04213 | 0.03786 | 0.04047 |

References:

Gao, H., Camargo, E. A., Ubi, J. N., Duan, X., Zheng, G., Gao, S., and Yuan, Q. (2025) Stereoselective construction of chiral flavanones via enzymatic intramolecular C (sp3)-H activation. Org. Chem. Front.
